# Development of mass allelic exchange, a technique to enable sexual genetics in *Escherichia coli*

**DOI:** 10.1073/pnas.2105458119

**Published:** 2022-11-02

**Authors:** Varnica Khetrapal, Liyana Ayub Ow Yong, Sylvester J. M. Lim, Swaine L. Chen

**Affiliations:** ^a^National University Health System Infectious Diseases Translational Research Programme, Department of Medicine, Division of Infectious Diseases, Yong Loo Lin School of Medicine, National University of Singapore, Singapore 119074;; ^b^Laboratory of Bacterial Genomics, Genome Institute of Singapore, Singapore 138672

**Keywords:** bacterial genetics, urinary tract infection, *Escherichia coli*, bacterial diversity, genetic engineering

## Abstract

Despite dramatic advances in genomics, connecting genotypes to phenotypes is still challenging. Sexual genetics combined with linkage analysis is a powerful solution to this problem but generally unavailable in bacteria. We build upon a strong negative selection system to invent mass allelic exchange (MAE), which enables hybridization of arbitrary (including pathogenic) strains of *Escherichia coli*. MAE reimplements the natural phenomenon of random cross-overs, enabling classical linkage analysis. We demonstrate the utility of MAE with virulence-related gain-of-function screens, discovering that transfer of a single operon from a uropathogenic strain is sufficient for enabling a commensal *E. coli* to form large intracellular bacterial collections within bladder epithelial cells. MAE thus enables assaying natural allelic variation in *E. coli* (and potentially other bacteria), complementing existing loss-of-function genomic techniques.

## Significance

Sexual genetics is a powerful strategy for dissecting any biological process; however, it is not applicable to most bacteria. We have invented mass allelic exchange (MAE), which enables direct sexual hybridization between arbitrary strains of *Escherichia coli* (including pathogenic clinical isolates). Like natural sexual hybrids in other organisms, MAE hybrids have no markers or scars. The combination of wild-type strains as well as scarless recombinants differentiates MAE from other genome-engineering technologies. By enabling access to the extant species variation, MAE facilitates a general method for gain-of-function screens for phenotypes of interest, a valuable complement to commonly used loss-of-function strategies. MAE will likely be feasible to implement in other Enterobacteriaceae, which are of strong medical and biotechnological interest.

The discovery of reassortment of phenotypes between two parental strains of *Escherichia coli* led to the discovery of conjugation and the realization that bacteria had an analog of sexual recombination (1, 2). Further exploitation of this phenomenon contributed to our modern understanding that genes are physically distributed on a chromosome, enabling the creation of genetic maps and the use of linkage analysis to associate genes with phenotypes (3). Since then, an explosion of genetic techniques have been developed to elucidate the genetic basis of myriad phenotypes. Interestingly, the use of sexual genetics has somewhat faded from modern bacteriology.

A dramatic recent example of the power of a sexual genetic strategy, even in (or enabled by) the postgenomic era, was the discovery that mutations within a single gene, *porA*, in *Campylobacter jejuni* were responsible for the ability of one clone to cause abortion in sheep (4). Leveraging the natural competence of *C. jejuni*, the genomic DNA from an abortifacient clone was incorporated into a nonabortifacient clone, generating a library of strains with distinct chromosomal hybridizations. A single infection experiment, coupled with whole-genome sequencing, led to the identification of *porA* as the causative locus for abortifacience out of a potential set of >8,000 single-nucleotide polymorphisms (SNPs) that differentiated the parental strains (4).

Thus, a sexual genetics strategy, coupled with the current widespread availability of genome sequencing, could be a powerful addition to our arsenal of genetic techniques, but this is currently practically limited to naturally competent bacteria, which encompass at least 80 species across both gram-positive and -negative bacteria (5, 6). In particular, sexual genetics allows a facile route to gain-of-function experiments that explore naturally occurring genetic diversity, which would complement strategies such as random mutagenesis or transposon screens (including transposon sequencing [Tn-seq]) that largely implement loss-of-function tests. While modifications to Tn-seq (with outward-facing promoters) (7, 8) or use of plasmid-based overexpression libraries can partially enable gain-of-function screens, these raise additional issues such as artificial expression levels and regulation.

Several advanced techniques have been recently developed to perform chromosome-wide engineering at a scale comparable to that achieved by the original Hfr-mediated *E. coli* recombinants. These include CAGE, MAGE, REXER, GENESIS, and REGRES (9–12); the power of REXER and GENESIS has been recently demonstrated with complete recoding of the *E. coli* genome to remove all instances of three codons, as a precursor to their repurposing for other synthetic biology applications (13). However, all of these technologies, except for REGRES, are designed for engineering defined chromosomal edits, albeit at the megabase scale. Furthermore, chromosome-wide engineering requires high efficiency, which has usually only been demonstrated in well-characterized (and “well-behaved”) cloning strains of *E. coli*. The REGRES technique, in contrast, achieves genotype–phenotype linkage similar in concept to the original Hfr technique. By leveraging a phenotypic difference between two strains, multiple rounds of conjugation are used to map the genetic locus responsible for the phenotype. This technique is a targeted screen requiring engineering and mating of strains during each round (11).

Here, we developed a system we term MAE (mass allelic exchange) that can generate libraries of hybrids for two parental *E. coli* strains in a single experiment. MAE leverages dedicated toxin genes as negative selection markers (instead of positive selection for antibiotic cassettes), which have been shown to be efficient in unmodified clinical strains (14), resulting in unmarked hybrid progeny and expanding the range of application to arbitrary strains of *E. coli* (and, likely, other Enterobacteriaceae). We demonstrate the efficiency of MAE by creating an arrayed library that includes hybrids covering >95% of the *E. coli* chromosome. Finally, we show the utility of MAE libraries by identifying a single operon, *chu*, from a uropathogenic strain of *E. coli*, transfer of which enables a commensal strain of *E. coli* to infect and replicate to high numbers within bladder epithelial cells.

## Results and Discussion

### Design and Optimization of MAE.

**Fig. 1.**
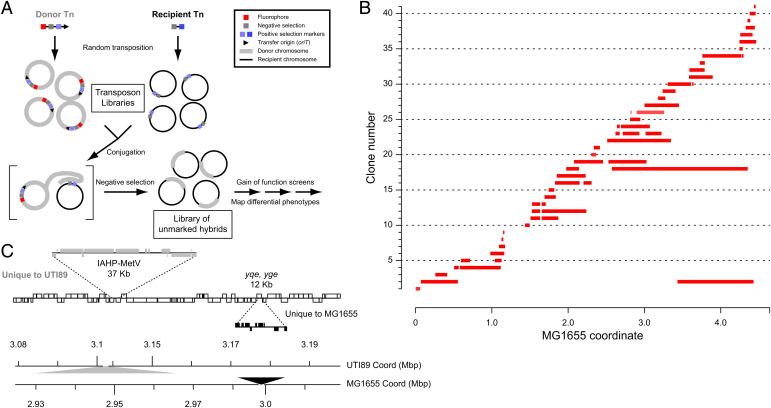
Overview of MAE. (*A*) Schematic of the MAE protocol. See text for further details. Each circle represents a single bacterial chromosome within a cell (for clarity, cell membranes are not drawn). Genetic markers are represented by colored squares, according to the legend at the top right. The fluorescent protein used was mCherry (red box). The negative selection module contained the *tse2* toxin gene under the control of P*rhaB* promoter (for both the donor and recipient transposon libraries, gray box). A kanamycin resistance gene (light blue box) was used as the positive selection marker for the donor libraries and a chloramphenicol resistance gene (dark blue box) was used for the recipient libraries. Experimental manipulations are indicated by the text adjacent to arrows. Text boxes indicate libraries of clones. Brackets enclose a representative single donor and recipient conjugation in the bottom left section. (*B*) Transferred SNPs for the SLC-H1 library of hybrids. Sequencing data from individual transconjugants were analyzed using the MG1655 genome as a reference. Genomic coordinate is plotted on the *x* axis. Individual clones are plotted at different locations on the *y* axis. Blocks of consecutive SNPs relative to the MG1655 genome sequence are represented by red bars. (*C*) One transconjugant was assembled and compared to the UTI89 and MG1655 genome reference sequences. A comparison of the annotations of ∼100 kb of homologous regions of the UTI89 and MG1655 chromosomes is shown. Annotated genes are indicated by rectangles in the upper graph; shared genes are indicated by open rectangles, with coding strand indicated by position of the rectangle above (positive) or below (negative) the central line. Genes found only in UTI89 are indicated by gray rectangles and offset upwards. Genes found only in MG1655 are indicated by black rectangles and offset downward. The genomic coordinates for each reference are shown on the dual scale at the bottom. Filled gray (for UTI89) and black (for MG1655) triangles indicate the location and length of large DNA insertions relative to the other strain. In the ∼100-kb region depicted, the transconjugant matches the UTI89 sequence, with assembled sequence matching the IAHP-MetV pathogenicity island but not the *yqe*, *yge* island.

A conceptual schematic of MAE is shown in Fig. 1*A*. We designate one *E. coli* strain as the donor and the other as the recipient. In this study the uropathogenic strain UTI89 is the donor while the nonpathogenic strain MG1655 is the recipient. Both strains are subjected to a Tn5-mediated transposon mutagenesis with a customized transposon which carries a stringent negative selection cassette [P*_rhaB_*-*tse2*, i.e., the toxic *tse2* gene under the control of a rhamnose-inducible promoter (14)]. The donor transposon additionally has an outward facing *oriT* (origin of transfer) sequence to initiate chromosomal transfer adjacent to (and not including) the custom transposon. For tracking efficiency, the donor and recipient transposons also differ in their positively selectable antibiotic markers and a fluorescent protein (kanamycin resistance and mCherry fluorescence on the donor transposon, chloramphenicol resistance on the recipient transposon, and a plasmid-based green fluorescent protein [GFP] fluorescence in recipients). Finally, the donor is transformed with a helper plasmid for conjugation [pRK24 (9)], then a standard conjugation is performed. Selection of the mixture on restrictive conditions for the negative selection cassette (i.e., growth on minimal media M9 with rhamnose to induce expression of the toxic *tse2* gene) results in survival only of hybrid transconjugant strains; the donor negative selection cassette is not transferred due to the position and orientation of the *oriT*, while the recipient negative selection cassette is removed by recombination with incoming donor DNA. Of note, we had initially considered using transformation of purified genomic DNA (as was done with *C. jejuni*) or generalized transduction as methods to exchange DNA between strains; using these, the donor strain would not need to be modified. However, transformation was found to be too inefficient by several orders of magnitude, while transduction required strain-specific phages that are not generally available for many *E. coli* strains, especially clinical isolates (15).

The key innovation of MAE is the use of a stringent negative selection cassette (14), which has enough selective power to nearly quantitatively eliminate the numerically dominant, unhybridized donor and recipient cells; in a single step, we can thereby generate unmarked hybrids, which sets MAE apart from all previous technologies. Therefore, a practical issue limiting MAE was an apparent loss of negative selection stringency when using transposon libraries (10^−4^) instead of targeted insertions (10^−7^ to 10^−8^) (*SI Appendix*, Fig. S1*A*). Growth under restrictive conditions (M9 + rhamnose) of strains carrying the negative selection cassette can occur for multiple reasons, such as spontaneous mutation of the selection cassette, inactivation via insertion sequences or other mobile elements, or spontaneous deletion. Certain regions of the chromosome are known to have a high effective spontaneous deletion rate, such as prophages, pathogenicity islands, and plasmids (16, 17) (*SI Appendix*, Fig. S1*A*). We therefore solved this issue by screening individual colonies for their frequency of growth on restrictive conditions. Aggregation of the “stable” donor and recipient colonies (with an inactivation frequency of <10^−6^) then resulted in a donor and recipient library with an overall negative selection stringency similar to that previously reported (10^−7^) (*SI Appendix*, Supplementary Text 1). Of note, this process secondarily resulted in a rapid screen for inherently unstable regions of the *E. coli* chromosome; as expected, prophages, pathogenicity islands, and the plasmid are lost at relatively high frequency. Ribosomal RNA loci, which are known to undergo intrachromosomal gene conversion (18), were also found. In addition, we discovered previously unknown unstable regions that may provide new insights into mechanisms of chromosome plasticity, maintenance, and evolution (*SI Appendix*, Supplementary Text 1 and Fig. S1*B*).

### MAE Generates Libraries of Genome-Wide *E. coli* Hybrids in a Single Experiment.

In a single hybridization experiment we were able to generate thousands of hybrid transconjugants (based on blocks of SNPs transferred, denoted as red bars in *SI Appendix*, Fig. S2*A*). Based on resistance phenotypes, fluorescent markers, and whole-genome sequencing, we estimate that this pooled MAE library is composed of >98% true hybrid transconjugants. We saw a bias toward DNA exchange near the origin of replication (*SI Appendix*, Fig. S2*A*); this suggested that DNA replication, and the attendant differences in copy number between the origin and the terminus, was responsible (19). Indeed, we saw typical (3 to 7×) biases in the density of transposon insertions around the origin in the source donor and recipient libraries (*SI Appendix*, Fig. S2 *B* and *C*); conjugation and sequencing then incur additional bias amplifying origin-proximal transposon insertions and the resulting hybrids (*SI Appendix*, Fig. S2*A*). A quantitative analysis of the bias across both source libraries and the output transconjugant library indicated that the origin copy number effect might be sufficient to account for all of the observed bias (*SI Appendix*, Supplementary Text 2). To verify that terminus-proximal cross-overs were not further selected against, we performed directed transfers using individual donor and recipient strains, which showed no difference in efficiency (transconjugants per recipient) when the transfer was origin-proximal or terminus-proximal (*SI Appendix*, Fig. S2 *D* and *E*). In other words, the actual hybridization step in MAE introduced no additional bias. Therefore, control of growth rate (to limit multifork replication) and/or selection of uniformly (or terminus-biased) clones for the source libraries would effectively eliminate this bias. Alternatively, clone selection could be done after hybridization, particularly to create arrayed libraries useful for many genetic screens. We created the first such arrayed library of UTI89-MG1655 hybrids (SLC-H1), in which 96% of the MG1655 chromosome has been replaced by homologous sequence from UTI89 in at least one clone (the largest missing block being 146 kbp [3.15% of the genome]) (Fig. 1*B*).

All mutation types were accessible to transfer by MAE. Whole-genome sequencing demonstrated continuous tracts of donor SNPs being transferred to the homologous region of the recipient chromosome. These were full replacements of segments of the chromosome, ranging from 16 to 1,780 kb (median 298 kb) (Fig. 1*B* and *SI Appendix*, Fig. S2*A* and Supplementary Text 3), and they transferred all expected gene insertions and deletions as well as pathogenicity islands up to 80 kb (Fig. 1*C*). Most (267/353, 75.6%) hybrids had only one single block of DNA transferred, with additional separate blocks progressively more rare (Fig. 1*B* and *SI Appendix*, Figs. S2*A* and S3).

**Fig. 2.**
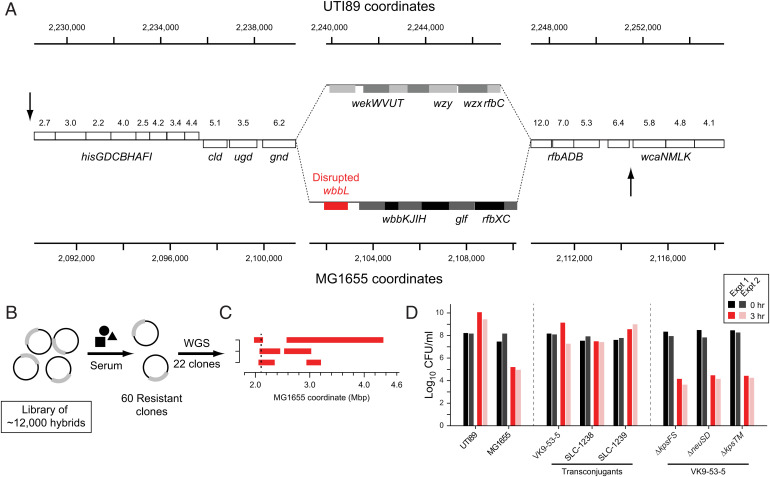
In vitro screening of MAE Libraries. (*A*) *rfb* locus comparison. Genome coordinates for UTI89 and MG1655 are shown on the axes at the top and bottom, respectively. Genes indicated by open boxes are nearly identical in sequence between UTI89 and MG1655; the percent nucleotide divergence is indicated by the numbers above each gene. Genes in the center section (gray boxes and red box) are not obviously homologous, with no sequence above 70% nucleotide identity in the other genome. Arrows indicate locations of donor (top arrow pointing down) and recipient (bottom arrow pointing up) insertions for targeted transfer. (*B*) Schematic of the serum and phage resistance screens. Each black circle represents a single bacterial chromosome within a cell (for clarity, cell membranes are not drawn). Experimental manipulations are indicated by the text adjacent to arrows. Thicker gray lines represent donor DNA sequence recombined into the recipient chromosome. (*C*) Twenty-two serum-resistant clones were sequenced, which were represented by three independent clones. The transferred regions for each clone are shown in red, based on the MG1655 coordinates (*x* axis). The two vertical black dotted lines (very closely spaced) indicate the region of the *rfb* locus from the disrupted *wbbL* gene to the *rfbXC* genes. (*D*) Quantification of phage resistance. Each strain was titered before (0 h, black and gray bars) and 3 h after (3 h, red and pink bars) mixing with P1 phage; the logarithm of the titers is plotted on the *x* axis. UTI89 is a resistant control (resistant to P1); MG1655 is a sensitive control. Strain VK-9-53-5 is a phage resistant transconjugant isolated from an MAE library; SLC-1238 and SLC-1239 are two transconjugant strains derived from a directed transfer near the *kps* locus. The right three lanes show mutants of VK9-53-5 with the indicated genes (all known to be required for capsule production) knocked out. All strains were run together in two separate experiments on different days.

One of the key advantages of allelic exchange methods over the popular Tn-seq or other random mutagenesis methods is the ability to select for phenotypes that differ between strains, particularly in a gain-of-function strategy. We used pooled MAE libraries to perform two such screens. The donor strain, UTI89, survives in human serum, resists P1 phage infection, and hemolyzes sheep blood, while the recipient strain, MG1655, lacks all of these phenotypes. The first two phenotypes are known to be due to an insertion sequence disrupting the MG1655 *wbbL* gene, which abolishes lipopolysaccharide O-antigen synthesis (20). Interestingly, the UTI89 *rfb* locus is organized differently from the corresponding MG1655 locus; restoration of O-antigen in MG1655 should therefore require replacement of the entire 10-kb locus (Fig. 2*A*). We first screened the pooled library for serum resistance, isolating 60 clones (Fig. 2*B* and *SI Appendix*, Fig. S4*A*). Whole-genome sequencing of 22 of these clones showed that only 3 independent transconjugants were isolated; in these, while the majority of the chromosome was indeed MG1655, 15- to 50-kb sections of the UTI89 genome encompassing the *rfb* locus were now integrated into the homologous location of MG1655 (Fig. 2*C*). As further confirmation, these clones had also gained resistance to P1 phage, as expected (*SI Appendix*, Fig. S4*B*). Finally, we verified separately that a targeted transfer of the entire *rfb* locus from UTI89 to MG1655 indeed is sufficient to confer resistance to serum and P1 phage infection (*SI Appendix*, Fig. S5).

Attesting to the ability of gain-of-function screens to provide complementary information, MAE screens provided new insight into these well-studied phenotypes. In control experiments to prepare for an intracellular infection screen (see below), we isolated phage-resistant clones in which the *rfb* locus had not been transferred; instead, a region of the chromosome 1 Mbp away, encompassing a UTI89-specific 30-kb island containing the *kps* and *neu* operons encoding a capsule biosynthetic pathway, had been transferred (VK9-53-5). Targeted transfer of this *kps*-containing locus (represented by the two distinct clones SLC-1238 and SLC-1239) showed that it does indeed confer an intermediate level of phage resistance (i.e., resistance only when infected with <10^10^ plaque-forming units per mL of P1 phage) (Fig. 2*D*). To verify that this was due to the capsule genes, we made several targeted deletions in the original transconjugant (VK9-53-5) within the *kps* operon, all of which have been previously shown to abolish capsule production; these led to high phage sensitivity in the originally isolated resistant transconjugant (Fig. 2*D*). This demonstrates that modification of the capsule, in the absence of restoration of O-antigen, can confer partial resistance to P1 phage in MG1655.

### MAE Libraries Identify the *chu* Operon as Genetically Sufficient for Formation of Intracellular Bacterial Structures, a Hallmark of Uropathogenic *E. coli*.

**Fig. 3.**
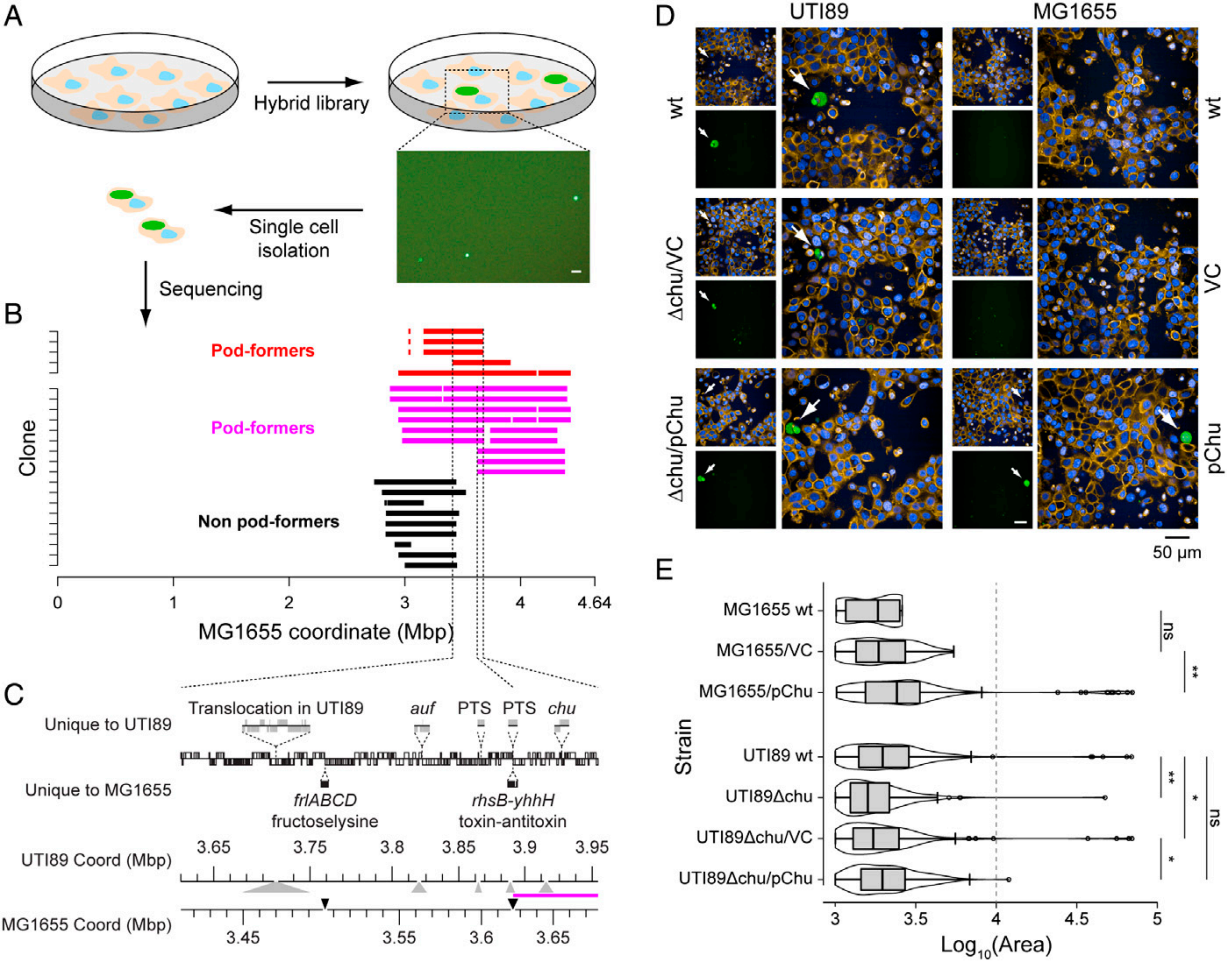
A gain-of-function screen for in vitro pod formation. (*A*) Schematic of the screen. Orange shapes represent individual bladder epithelial cells, light blue represents nuclei, and green represents aggregates of fluorescent *E. coli*. In the actual screen, 5637 cells at ∼10^6^ cells per well in a 6- or 12-well plate are infected with *E. coli* (either a transconjugant library or individual strains) at an MOI of 10 for 2 h, then a high-dose (100 μg/mL for 2 h) and a low-dose (10 μg/mL overnight) gentamycin treatment is used to kill extracellular bacteria; then, gentamycin is removed, 0.025% saponin is added for 30 min then washed away, and cells are observed for pod formation. A representative image of green fluorescence (*E. coli*) 6 h after saponin treatment and removal is shown at the bottom right of the panel. (Scale bar, 100 μm.) (*B*) Whole-genome sequencing of 5 pod-forming transconjugants (red) and 18 additional transconjugants (magenta and black; see text for details) (*y* axis). The transferred regions for each clone are shown in red or magenta (pod-forming transconjugants) or black (non-pod-forming transconjugants), based on the MG1655 coordinates (*x* axis). The left-most and right-most vertical dotted lines indicate the common region of UTI89 transferred to the clones depicted in red. The middle vertical dotted line delimits the left border of the common region of UTI89 transferred to all pod-forming transconjugants. (*C*) Genetic map of the common overlapping region for all pod-forming transconjugants. Notations are as in Fig. 1*C*. Magenta line between the UTI89 and MG1655 coordinate axes at the bottom denotes the common overlapping region of all pod-forming clones. (*D*) Representative images of the pod-forming assay. Each strain is represented by three images; the smaller images on the top are the DAPI (blue) and wheat germ agglutinin (orange) channels (*Left*) and GFP (green) (*Right*). The larger image is a merge of all channels. White arrows indicate identified large pods. Scale bar shown at the bottom is 50 μm for larger images. Scale bar (50 μm) for smaller images is in white in the MG1655/pChu GFP image. (*E*) Box-and-whisker plot of area (in pixels) for all intracellular structures identified for UTI89 strains. Log (base 10) of the area is indicated on the *x* axis, strain genotype on the *y* axis. Center line for each plot represents the median, boxes indicate the first and third quartiles, and whiskers are set at 1.5× the interquartile range. Open circles represent outliers outside the range of the whiskers. A violin plot is superimposed on each box, depicting the distribution of all the data. The vertical dotted line indicates 10,000 pixels. ns, *P* > 0.05; *P < 0.01; **P < 0.001 (two-tailed Wilcoxon rank-sum test).

Finally, we used MAE to explore a suspected complex phenotype: intracellular infection of cultured human epithelial cells. UTI89 is a uropathogenic strain; it robustly infects the bladder of mice and forms intracellular bacterial communities (IBCs) during acute phases of infection (21). IBC formation imposes a severe population bottleneck (> 10^4^ reduction) on the inoculated UTI89 (22). This bottleneck precludes the use of many genetic techniques to probe the mechanism of intracellular infection; therefore, several attempts have been made to create similar infection systems using in vitro–cultured cells (23, 24). One of these utilizes saponin treatment of infected bladder epithelial cells (5637 cells). In this system, UTI89 forms large intracellular aggregates of bacteria that are morphologically reminiscent of IBCs; these in vitro aggregates are referred to as pods. MG1655, in contrast, does not infect mouse bladders and also does not form pods in vitro (23). Therefore, infection of 5637 cells and treatment with saponin can be used as a gain-of-function screen for pod formation (Fig. 3*A*). We infected ∼10^6^ epithelial cells per well in a six-well plate at a multiplicity of infection (MOI) of 10 and found that pods were formed when we used our MAE library. We isolated five individual pod-containing cells from two different experiments; whole-genome sequencing revealed a common 314-kb region of the UTI89 chromosome that had been transferred in all clones (Fig. 3*B*, red clones). This region contained 6,148 SNPs, 35 genes within 4 operons present in UTI89 but not MG1655, another island of 34 translocated genes (i.e., they are also present in MG1655 but at a different chromosomal location), and 3 genes within 1 operon present in MG1655 but not in UTI89 (Fig. 3*C*). To further map this region, we screened another 102 individual transconjugants using PCRs for six genes within this 314-kb region that were present only in UTI89 or MG1655. Eighteen transconjugants were found that appeared to have transferred at least some of the 314-kb locus (with six having transferred all). These 18 transconjugants were also tested for pod formation; 9 formed pods, while the remaining 9 did not. All 18 of these were sequenced (Fig. 3B, magenta and black); only 36 kb was now shared among all pod-forming clones. This 36-kb pod-associated locus contained a total of 20 genes shared between UTI89 and MG1655, with average nucleotide identity of 96.9%, and two operons (encoding a PTS system and the *chu* heme acquisition system) that were present only in UTI89.

We first tested the hypothesis that the UTI89-specific operons were sufficient for conferring pod formation to MG1655. We transformed plasmids carrying each of these operons into MG1655; only the strain carrying the UTI89 *chu* operon was able to form large pods (Fig. 3 *D* and *E*, Movie S1, and *SI Appendix*, Fig. S6*A*); note that we saw high variability in pod formation between experiments for the control strain (i.e., only a subset of experiments demonstrated large pod formation; *SI Appendix*, Fig. S6 *B* and *C*), as was also noted by the laboratory that originally reported this phenotype (for details on the analysis in the face of this variability see *SI Appendix*, Supplementary Text 6). In addition, deletion of the *chu* operon (but not the *auf* and the *PTS* operon) from the transconjugant clones eliminated large pods, a qualitative phenotype that could be restored by recomplementation of the *chu* operon on a plasmid (*SI Appendix*, Fig. S6 *D–F*). Consistent with previous reports examining IBCs in mouse bladders (25), deletion of the *chu* operon in UTI89 led to smaller intracellular structures formed by UTI89 overall (Fig. 3*E*, Movie S2, and *SI Appendix*, Fig. S6 *D–F*), although large pods were still able to be formed (again with high variability between experiments). In contrast, MG1655 strictly required the presence of the *chu* operon to form such pods. We therefore confirm previous results that the *chu* operon is not necessary in UTI89 for formation of large intracellular structures (25); however, we now further demonstrate that the *chu* operon is sufficient to confer pod formation to MG1655.

Notably, MAE libraries coupled with this gain-of-function screening strategy were critical to make this discovery of the sufficiency of the *chu* operon. Previous gene expression– and loss-of-function-based strategies had identified this locus playing a quantitative but not necessary role in intracellular infection in UTI89 (25). Perhaps because many other loci (prominent among which is the *fim* operon) are indeed necessary for intracellular infection (based on loss-of-function experiments), no further studies have examined the role of the *chu* operon in intracellular infection. The additional genetic information about the sufficiency of the *chu* operon therefore provides a key complementary insight into the mechanism of intracellular infection, which may focus future research efforts.

## Conclusion

In summary, we have created intentional, unmarked whole-genome hybrids, akin to F1 progeny, between two distinct strains of *E. coli*, in a process we term MAE. The core enabling technology for MAE is the negative selection system, which provides high selective headroom and is usable in all tested *E. coli* strains, as well as in *Klebsiella*, *Salmonella* (14), *Serratia marcescens*, *Shigella flexneri, Enterobacter cloacae* (26), and *Providencia stuartii* (27), including clinical isolates. Thus, combined with existing methods for Hfr conjugation, transposon mutagenesis, and homologous recombination, MAE should be further accessible to bacteria besides *E. coli*, particularly other Enterobacteriaceae. The modular nature of the key negative selection cassette provides a straightforward potential route to generalization to other organisms as well.

There are some limitations to MAE. An ability to perform transposon mutagenesis and conjugation in the parental strains of interest is required. However, because MAE results in transfer of large chromosomal segments (median 298 kb) with high variability (*SI Appendix*, Supplementary Text 3), the whole-genome MAE libraries could be effectively constructed with limited numbers of donor and recipient clones, thus reducing the demand for full optimization of transposition and conjugation conditions and the labor in identifying stable transposon insertions. Fortunately, the critical negative selection cassettes have been shown in multiple species and strains to function well without the need for optimizing selection conditions (14). The transfer of large segments in turn raises issues for the resolution obtained in an MAE screen (i.e., how small the identified genetic locus is). Finally, as MAE generates unmarked hybrids, assessment of any results effectively requires whole-genome sequencing. These latter two issues may be mitigated by the combination of being able to generate large pooled libraries of MAE clones and continued advances in sequencing technology, making it easier to create and analyze experiments with more complex pools. Regardless, MAE is most unique for its ability to enable large-scale testing of all mutation types in both gain- and loss-of-function strategies (discussed further below); thus, as with all new technologies, MAE will be most powerful when combined cleverly with other existing methods that may be better suited to isolating and manipulating single genes.

As with traditional sexual genetics, MAE provides access to all types of genetic variation between strains, from SNPs to large (>100 kb) chromosomal insertions and deletions. Furthermore, MAE tests existing wild-type sequences, as opposed to artificial deletions or disruptions, and there are no residual genetic scars or markers in the resulting hybrid strains; the latter in particular are distinct advantages over other strategies such as using cosmid or BAC libraries, which may be further complicated by copy number differences, additional selection cassettes, and differences in chromosomal context. Gain-of-function experiments are a natural application area for MAE, which complements other techniques that enable loss-of-function experiments. While the power of sexual genetics has long been accessible in naturally competent bacteria such as *Campylobacter*, *Vibrio*, *Streptococcus*, and *Bacillus* spp. (5, 6), MAE now expands its reach to additional nonnaturally competent species. Moreover, MAE as we have implemented it results mostly in transfer of single, large chromosomal segments, which are substantially fewer and larger than those considered “large” for transfer by natural competence (28, 29), leading us to speculate that MAE may also be a useful complementary tool for naturally competent bacteria, particularly for phenotypes dependent on very large pathogenicity islands or multiple unlinked loci. In addition to providing insights into previously inaccessible (or difficult-to-access) complex phenotypes, MAE also provides an additional technology to support the directed evolution of bacterial strains, enabling strategies akin to breeding of other organisms (such as livestock or pets) for making customized chassis for biotechnology or synthetic biology applications.

## Materials and Methods

UTI89 and MG1655 were obtained from laboratory stocks originally derived from those in the laboratory of Scott Hultgren. Custom transposons were constructed from constituent components by PCR, mixed with purified Tn5 transposase (Epicentre) according to the manufacturer’s instructions, and transformed into UTI89 or MG1655. Selections were carried out on using the following concentrations of antibiotics (from Sigma-Aldrich Pte. Ltd.), as appropriate: ampicillin (100 μg/mL), kanamycin (50 μg/mL, for plasmid-based markers), 25 μg/mL, for chromosomal markers), chloramphenicol (20 μg/mL), tetracycline (10 μg/mL), streptomycin (50 μg/mL), and gentamycin (3 μg/mL). For negative selection markers, media was supplemented with glucose or rhamnose (Sigma-Aldrich, Pte Ltd.), as indicated, both at 0.2%. The 5637 bladder epithelial cells were obtained from ATCC (product HTB-9). Whole-genome sequencing libraries were prepared using either Illumina TruSeq DNA Nano library preparation kit (where maximum of 24 samples were multiplexed together) or Illumina Nextera DNA library prep kit (where 96 to 384 samples were multiplexed together) according to the manufacturer's recommendations. Sequencing was done on an Illumina HiSeq4000 or NextSeq with 2 × 151 bp reads depending on machine availability. Sequence analysis was done using ad hoc custom scripts. Additional experimental and analytical details can be found in *SI Appendix*.

## Supplementary Material

Supplementary File

Supplementary File

Supplementary File

## Data Availability

All study data are included in the article and/or supporting information.
